# Development of a droplet digital PCR assay to detect bovine alphaherpesvirus 1 in bovine semen

**DOI:** 10.1186/s12917-022-03235-2

**Published:** 2022-04-02

**Authors:** Zhichao Yu, Zhiguo Zhao, Linjun Chen, Han Yan, Qiang Cui, Xianghong Ju, Yanhong Yong, Xiaoxi Liu, Xingbin Ma, Guanhua Zhang

**Affiliations:** 1 Department of Veterinary Medicine, College of Coastal Agricultural Sciences, Guangdong Ocean University, Zhanjiang, 524088 Guangdong China; 2 Technology Center, Hohhot Customs District, Hohhot, 010020 Inner Mongolia China; 3 Comprehensive Inspection and Testing Center, Chifeng, 024000 Inner Mongolia China

**Keywords:** Bovine alphaherpesvirus 1, Real-time PCR, Droplet digital PCR, Bovine semen, Detection method

## Abstract

**Background:**

Infectious bovine rhinotracheitis (IBR) caused by bovine alphaherpesvirus 1 (BoHV-1) is one of the most important contagious diseases in bovine. This is one of the most common infectious disease of cattle. This has led to high economic losses in the cattle farming industry. BoHV-1 can potentially be transmitted via semen during natural or artificial insemination (AI). Therefore, testing methods for the early diagnosis of BoHV-1 infection are urgently needed for international trade of ruminant semen. In this study, we developed a novel droplet digital PCR (ddPCR) assay for the detection of BoHV-1 DNA in semen samples.

**Results:**

The ddPCR results showed that the detection limit was 4.45 copies per reaction with high reproducibility. The established method was highly specific for BoHV-1 and did not show cross-reactivity with specify the organisms (BTV, BVDV, *Brucella*, *M . bovis*). The results of clinical sample testing showed that the positivity rate of ddPCR (87.8%) was higher than that of qPCR (84.1%).

**Conclusions:**

The ddPCR assay showed good accuracy for mixed samples and could be a new added diagnostic tool for detecting BoHV-1.

**Supplementary Information:**

The online version contains supplementary material available at 10.1186/s12917-022-03235-2.

## Background

Infectious bovine rhinotracheitis (IBR) is caused by bovine alphaherpesvirus 1 (BoHV-1), which belongs to the family *Herpesviridae* and the genus *Varicellovirus* [1]*.* IBR is an acute, fever-inducing and highly contagious disease in cattle and buffalo and is characterized by general respiratory disease, conjunctivitis, balanoposthitis, encephalitis and miscarriage [2, 3]. The disease is responsible for considerable economic losses in animal production. It is also distributed worldwide and classified as a reported disease by the World Health Organization for Animals (OIE) [4, 5]. The virus is intermittently excreted in the semen of IBR-seropositive bulls, and the introduction of IBR may also occur through contaminated semen during natural or artificial insemination (AI) [6].

The testing of semen from bulls preceding admission to trade in animals and animal products is highly recommended, especially in international trade [7, 8]. The OIE Terrestrial Animal Health Code recommends screening of the bulls to be tested for the absence of BoHV-1 by either virus isolation or real-time PCR (qPCR) [9]. Unfortunately, laboratory diagnostic tests on semen have considerable limitations, such as the virucidal and cytotoxic properties of seminal plasma in cell culture and the long duration required for viral culture growth [10, 11]. Although the qPCR test is faster and specific, it requires the establishment of a standard curve for absolute quantification [12]. Additionally, the sensitivity of qPCR is limited when detecting mixed samples with low viral concentrations in large-scale examination of samples because the semen properties can lead to inhibition of transcriptase enzymes [13, 14]. Therefore, there is a need to establish a more sensitive assay.

To date, droplet digital PCR (ddPCR), as a novel nucleic acid detection technology, has enabled the absolute quantitation of nucleic acids without the need for a standard curve. ddPCR uses the same primers and probe as qPCR but can achieve high sensitivity [15, 16]. The reaction mixture is separated into a multitude of water-in-oil droplets, and a combination of microfluidic techniques is used to indicate the target gene via a Poisson distribution and analysis of the number of positive and negative partitions [17]. The ddPCR method has been successfully applied for the detection of viral and bacterial infections. The advantage of ddPCR is that it is highly sensitive and specific and improves the accuracy of target nucleic acid detection at low concentrations compared with qPCR [18, 19]. Accordingly, the aim of this study was to develop a ddPCR assay for the detection of BoHV-1 in semen samples of cattle. Moreover, we evaluated the sensitivity, specificity and repeatability of this method and the effectiveness of testing the semen samples.

## Materials and methodology

### Virus and semen samples

Inactivated BoHV-1 (NC_001847.1), bluetongue virus (BTV, MT119862.1) and bovine viral diarrhea virus (BVDV, AF091605.1) culture media were obtained from the Technology Center of KunMing Customs District. Serum *Brucella* (LT671512.1) and *Mycobacterium bovis* (CP064405.1) samples and bovine semen samples were obtained from the Technology Center of Hohhot Customs District. The positivity and negativity were determined according to the results of virus isolation. The bovine semen samples were frozen semen.

### Primers and probes design

The consensus sequence was selected to design primers and probes by comparing the BoHV-1 gB gene fragments (KY348790.1, AY745875.1, MF421714.1, DQ006855.1, JN787952.1, KJ652514.1, KF601565.1, JX127194.1, KU992438.1) obtained from GenBank. Sequence alignment was carried out using DNAMAN software (Lynnon Biosoft, USA). According to the analysis results, the primers and TaqMan probe were designed (Additional file 1: Table S1). All primers and probes were synthesized by Sangon Biotech (Shanghai, China).

### Nucleic acid extraction and reverse transcription

Nucleic acids were extracted from pathogen and semen samples using a MiniBEST Viral RNA/DNA Extraction Kit (TaKaRa Biotech Co., Ltd., Beijing, China). The volume of semen was 200 μL carried out following up kit instructions. Reverse transcription was carried out by incubating 10 μL of RNA with 4 μL of 5 × AMV buffer, 2 μL of dNTPs (2.5 mmol/L), 0.5 μL of RNasin (40 U/μL), 1 μL of random primer, and 1 μL of AMV reverse transcriptase (5 U/μL), and RNase-free H_2_O was added to obtain a final volume of 20 μL. The mixture was incubated at 42 °C for 60 min and then at 99 °C for 5 min.

### Preparation of cloned plasmid standards

The BoHV-1 gB gene fragments were separately amplified by PCR with the corresponding primer pair (Additional file 1: Table S1). The reaction was carried out using 2 × *EasyTaq* PCR SuperMix (TransGen Biotech Co., Ltd., Beijing, China). The PCR-amplified products were purified using a FastPure Gel DNA Extraction Mini Kit (Vazyme Biotech Co., Ltd., Jiangsu, China), the target gene was cloned into plasmid vectors, and the recombinant plasmid was purified using a FastPure Plasmid Kit (Vazyme Biotech Co., Ltd., Jiangsu, China). The plasmid was verified by DNA sequencing (Sangon Biotech, Shanghai, China), and the results were compared with the corresponding sequence data in GenBank. The concentration of the plasmid DNA was measured by a NanoDrop 2000 spectrophotometer (Thermo Scientific, USA) and recalculated to determine the plasmid copies/μL using a previously reported method [20]. The initial concentration of the BoHV-1 plasmid was 4.45 × 10^9^ copies/μL.

### qPCR method

The plasmid DNA was amplified using the primers and probe by qPCR. qPCR was carried out using the QuantStudio 6 Flex Real-time PCR System (Thermo Fisher Scientific, USA) and TransStart Probe qPCR SuperMix (TransGen Biotech Co., Ltd., Beijing, China). The standard curve was generated by using tenfold serial dilutions of standard plasmid DNA. The cycle threshold (Ct) value of the sample was used to calculate the copy number of each sample. The 25 μL qPCR reaction comprised 10 μL of 2 × TransStart Probe qPCR SuperMix, 5 μL of plasmid DNA, 1 μL of each of the primers, 0.8 μL of probe, and RNase-free H_2_O to reach the final volume. The reaction conditions for qPCR were as follows: 95 °C for 2 min, followed by 40 cycles of 95 °C for 15 s and 58 °C for 45 s.

### ddPCR method

The same primers and probe were used for ddPCR. The annealing temperature was varied for the ddPCR assay to determine the optimal conditions. The ddPCR assay was performed in a 20 μL reaction, containing 10 μL of 2 × ddPCR Supermix (Bio-Rad, Co., Ltd., California, USA), 1 μL of plasmid DNA, 1.6 μM (800 nmol/L) each of the primers, and 0.4 μL (200 nmol/L) of the probe. Next, the mixture was placed in a QX200 Droplet Generator (Bio-Rad, USA) to generate microdroplets according to the manufacturer’s instructions. Then, the droplets were transferred into a 96-well PCR plate, and the plate was sealed with foil by using a PX1 PCR Plate Sealer (Bio-Rad, USA). The amplification reaction protocol was as follows: 95 °C for 10 min, 40 cycles of 95 °C for 30 s and a temperature gradient from 56 to 62 °C for 1 min, and 98 °C for 5 min, ending at 16 °C, with a temperature variation rate of 2.5 °C/s. The contents of each well of the plate were removed and read by a QX200 Droplet Reader (Bio-Rad, USA), and the results were analyzed using QuantaSoft software (Bio-Rad, USA).

### Analytical sensitivity and repeatability

The plasmid DNA was serially diluted tenfold to concentrations ranging from 4.45 × 10^9^ to 4.45 × 10^0^ copies/μL, and the dilutions were used to determine the sensitivity separately by a qPCR assay. ddPCR was suitable for detecting low-concentration samples. Therefore, plasmid templates containing less than 10^4^ copies/μL were prepared.

### Analytical specificity

To separately determine the specificity of the established ddPCR method, important vertically transmitted cattle pathogens, including BTV, BVDV, *Brucella* and *M. bovis,* were tested.

### The clinical samples detection

To evaluate the efficiency of the established ddPCR for the single and mixed samples. A total of 50 bovine semen samples (40 BoHV-1-positive and 10 BoHV-1-negative) were single tested by the qPCR and ddPCR. Then, these positive and negative samples were randomly selected and mixed in different proportions (Additional file 1: Table S2), and these mixed samples were divided into the Nos. 1-4 groups (8 samples in each group). And a total of 32 mixed samples were tested by ddPCR and qPCR. In qPCR, Any sample that has a Ct value less than 40 is regarded as positive and more than 40 is regarded as suspicious. Any sample that shows no Ct value is regarded as negative.

## Results

### Optimal thermal gradient for ddPCR

**Fig. 1:**
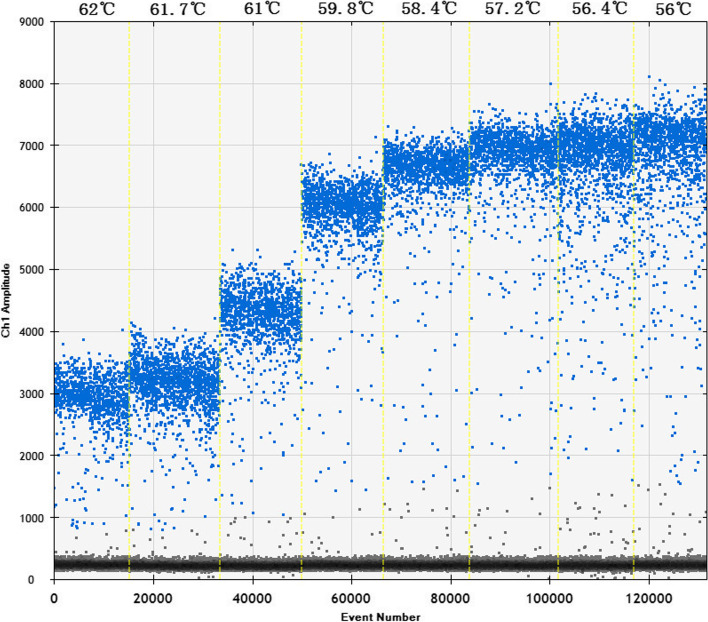
Optimization of the annealing temperature for the ddPCR method

The greatest difference between the fluorescence values of the positive (blue) and negative (gray) samples with the largest number of amplicons (positive droplets) was obtained at 57.2 °C; hence, this was selected as the optimal annealing temperature (Fig. 1).

### Analytical sensitivity and repeatability

**Fig. 2:**
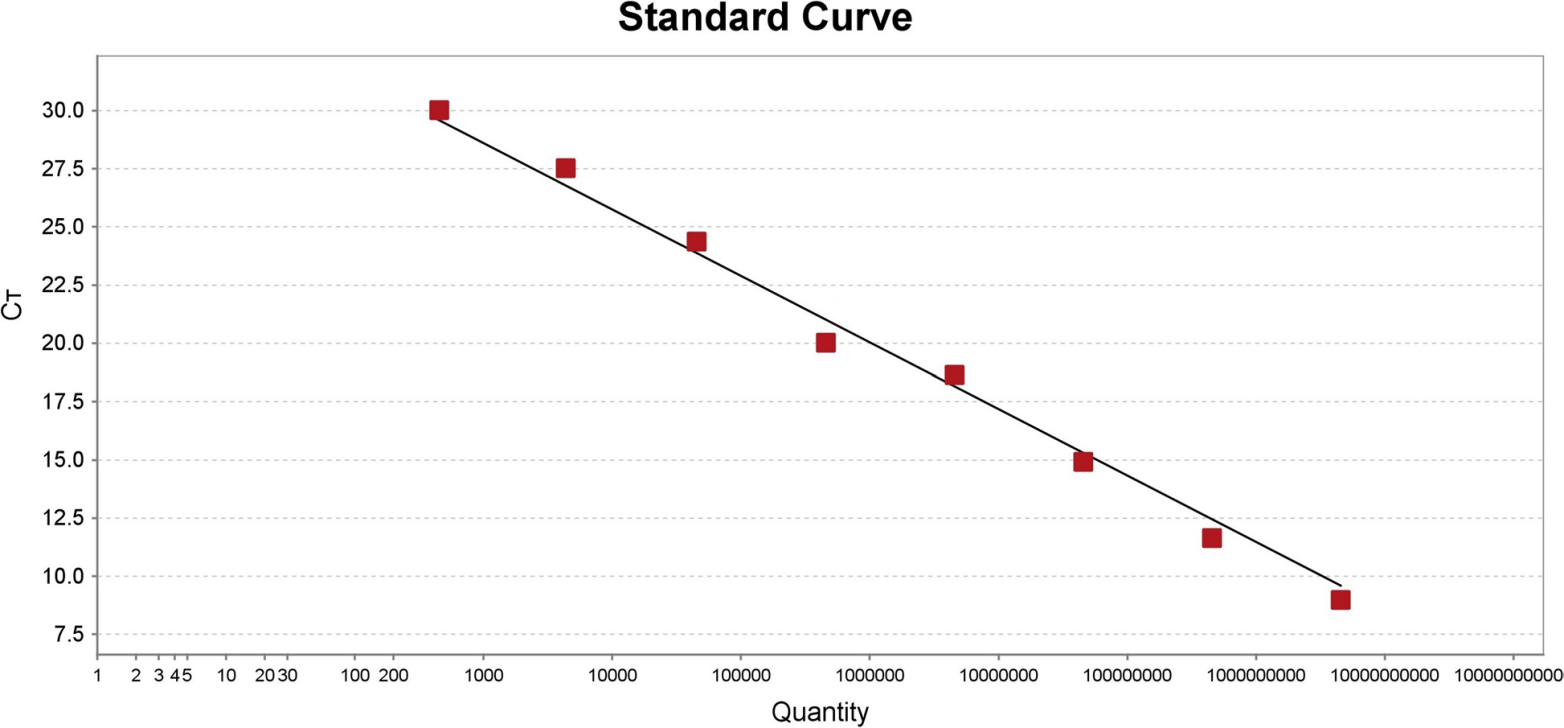
qPCR standard curve from a 10-fold dilution series of plasmid templates. The slope of the fitted line is − 2.85, *R*^2^ = 0.944

**Fig. 3:**
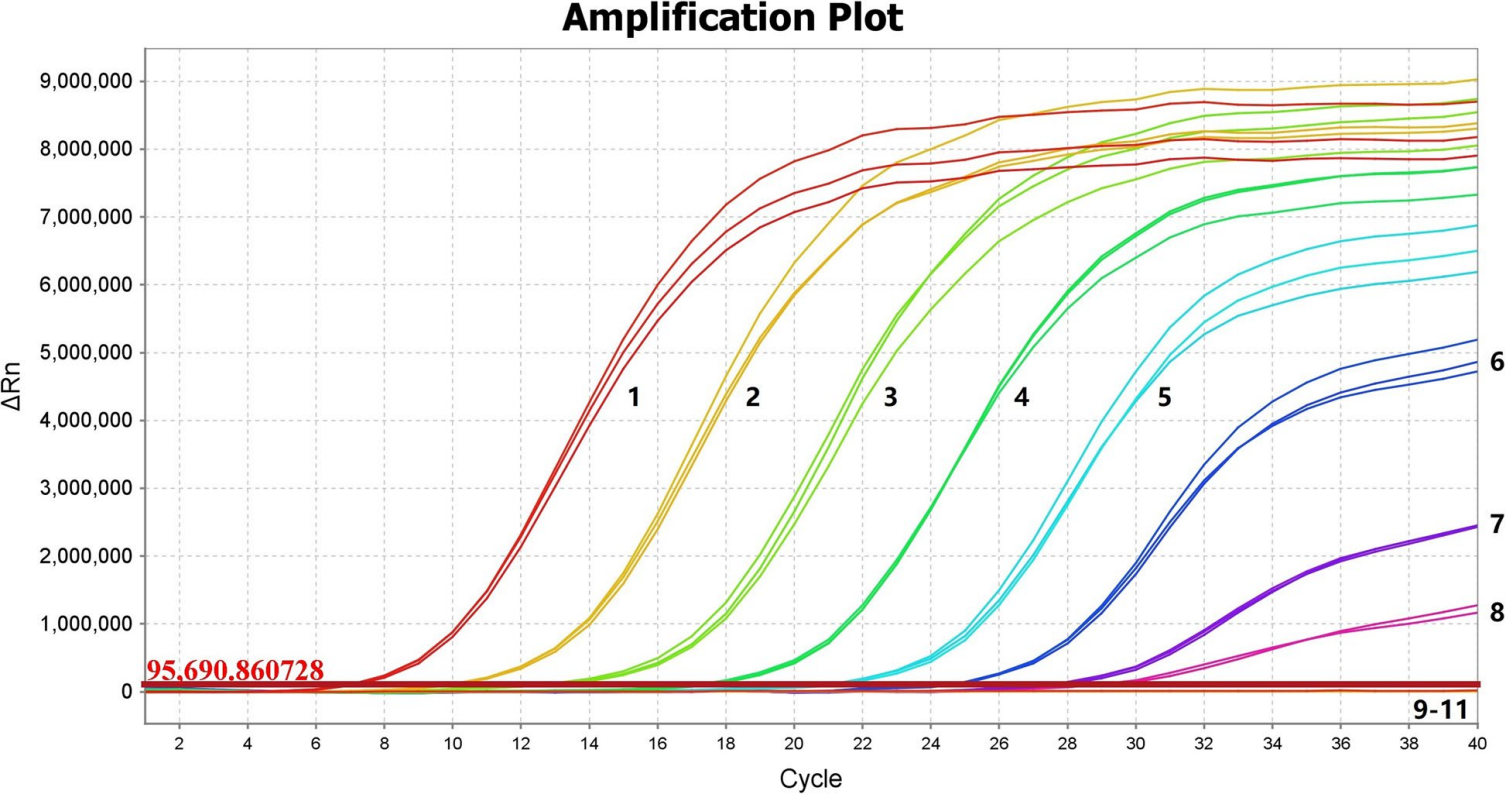
Sensitivity analysis of the amplification curve of the qPCR method. Limit of detection of qPCR from serial dilutions (4.45 × 10^9^ to 4.45 × 10^2^) of plasmid templates. 1-10: 4.45 × 10^9^-4.45 × 10^0^ copies/μL dilution standard. 11: Negative control

**Fig. 4:**
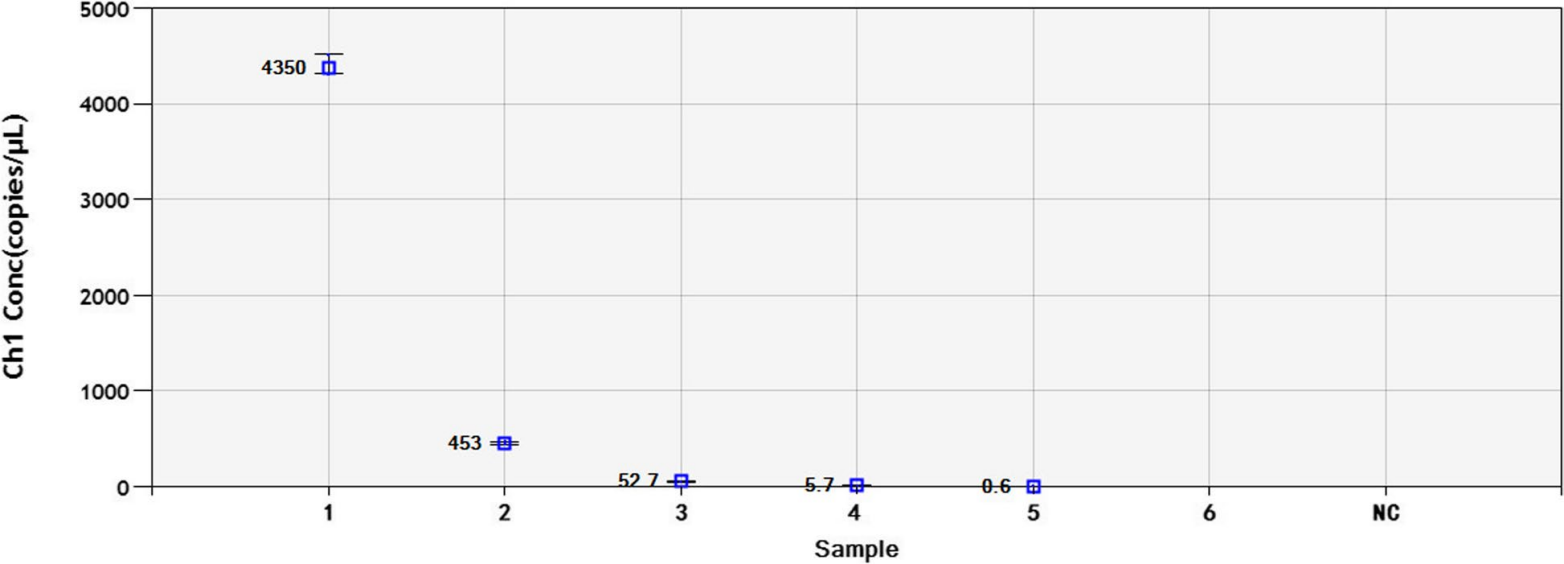
Sensitivity analysis of the ddPCR assay with different DNA dilutions. 1-6: 4.45 × 10^4^-4.45 × 10^− 1^ copies/μL dilution standard. NC: negative control

**Fig. 5:**
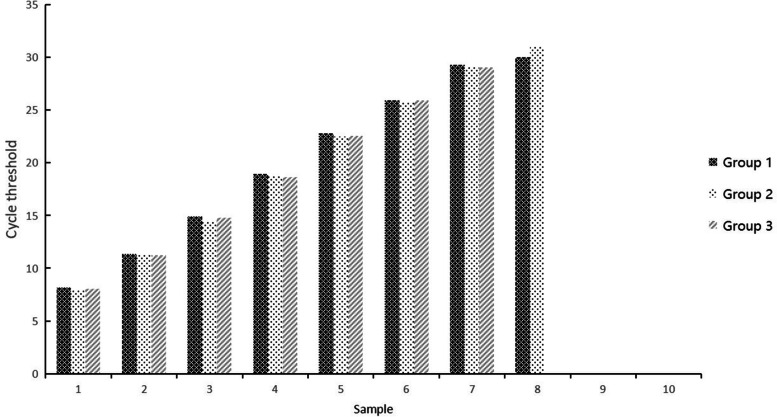
Sensitivity analysis of the qPCR assay with different DNA dilutions. 1-10: 4.45 × 10^9^-4.45 × 10^0^ copies/μL dilution standard

**Fig. 6:**
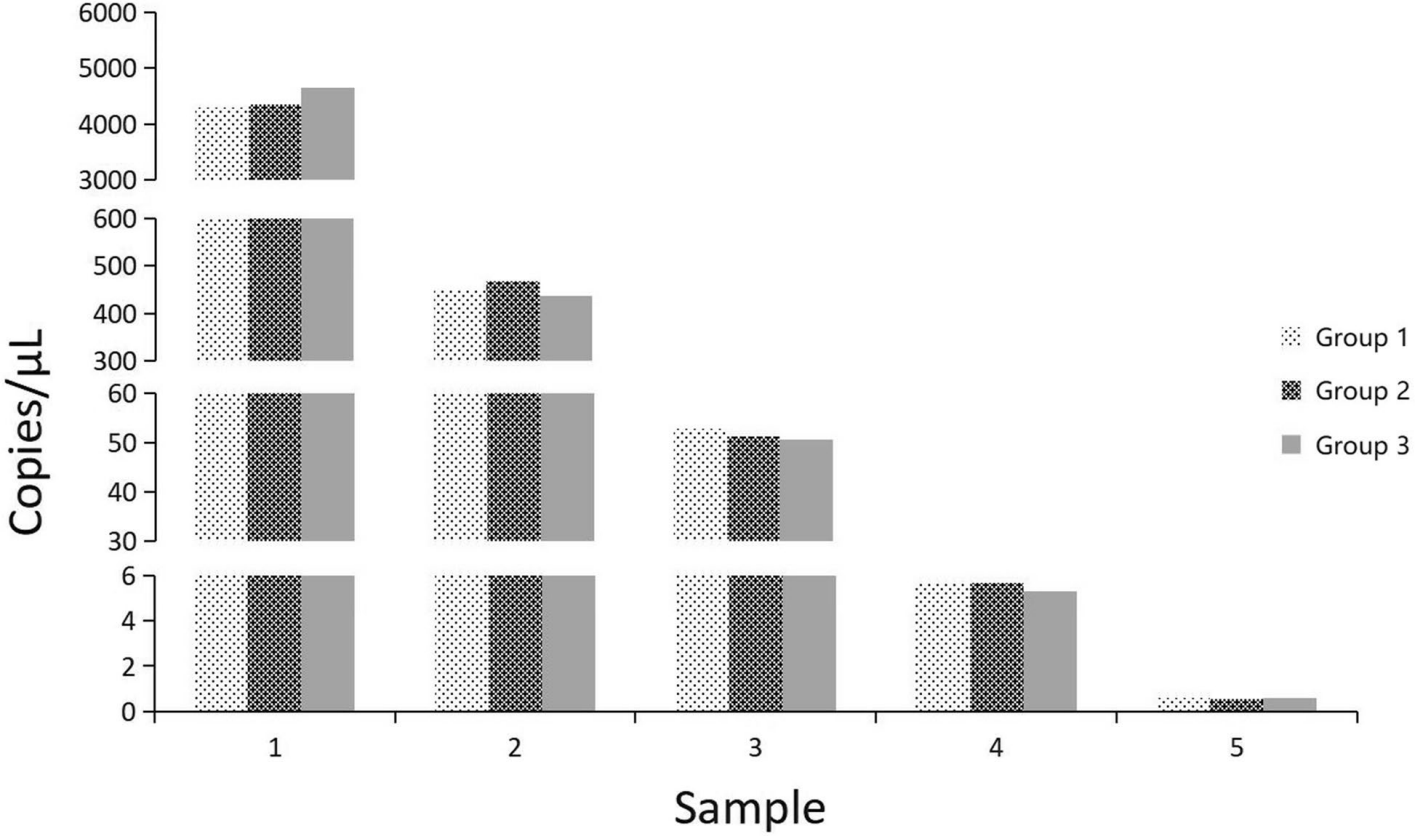
Repeatability analysis of the DNA concentration in each diluted sample determined by the ddPCR assay. 1-5: 4.45 × 10^4^-4.45 × 10^0^ copies/μL dilution standard

The serially diluted plasmids demonstrated good PCR efficiency in both the qPCR and ddPCR assays. In qPCR, the standard curve exhibited good linearity (y = − 2.85x + 37.16) with a correlation coefficient of *R*^2^ = 0.944 (Fig. 2); the lowest detection limit was 4.45 × 10^2^ copies/μL with poor repeatability, and concentrations below that dilutions were not detectable (Fig. 3). However, the limit for ddPCR was 4.45 copies per reaction, which was 100-fold lower than that for qPCR (Fig. 4). In the repeatability analysis (repeated three times), ddPCR showed good repeatability for detecting low-concentration samples in comparison with qPCR (Figs. 5-6).

### Analytical specificity of the ddPCR method

**Fig. 7:**
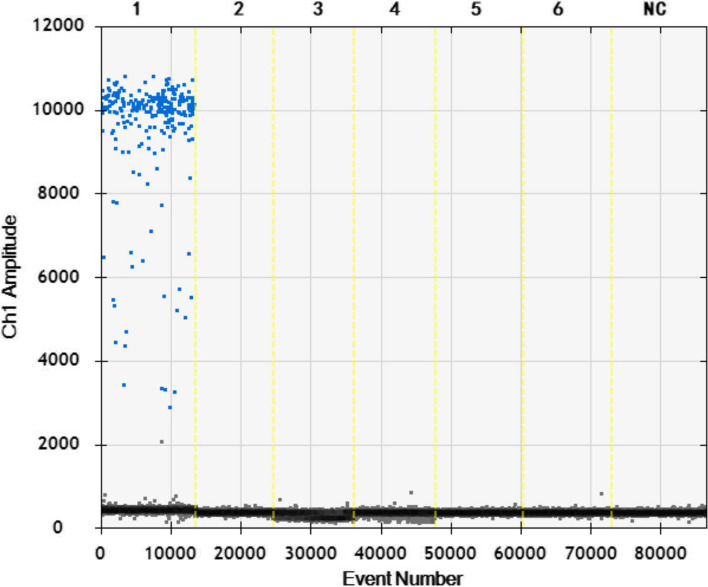
Specificity of the ddPCR assay. Lanes 1-6: BoHV-1 plasmid, BTV, BVDV, *Brucella* and *Mycobacterium bovis*. Lane NC: negative control

**Fig. 8:**
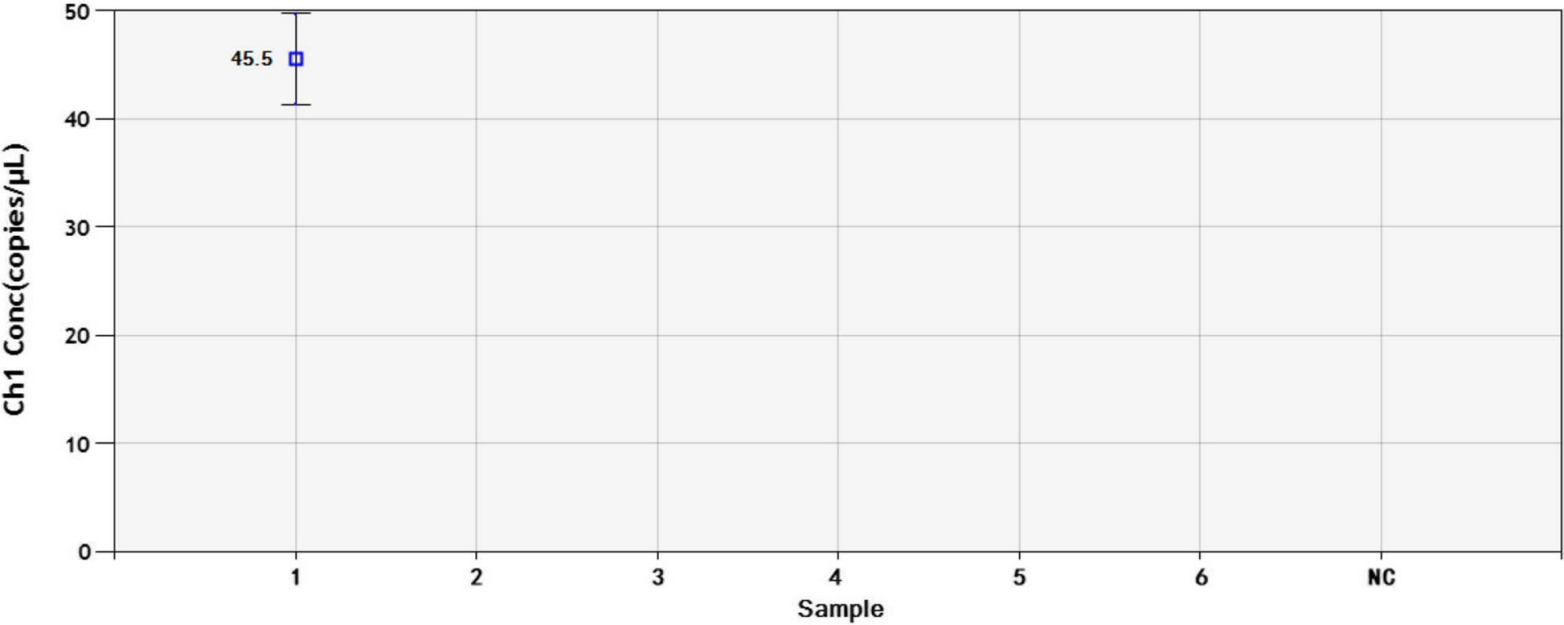
Specificity of the ddPCR assay. 1-6: BoHV-1 plasmid, BTV, BVDV, *Brucella* and *Mycobacterium bovis*. NC: negative control

For the specificity analysis, the DNA/cDNA of BTV, BVDV, *Brucella* and *M. bovis* were tested by the ddPCR assay. As shown in Figs. 7-8, only the BoHV-1 plasmid tested positive, while the other samples tested negative. The results indicated that this method is specific for the detection of BoHV-1.

### Evaluating the ddPCR assay with clinical samples

As shown in Table 1, the positivity rate of ddPCR (87.8%) was higher than that of qPCR (84.1%). 82 bovine semen samples (50 single and 32 mixed) were tested using qPCR and ddPCR. The detection results of both methods were accurate (40 BoHV-1-positive and 10 BoHV-1-negative). When the samples were mixed by groups, the results showed that Nos. 1 and 2 were all positive, while the positivity rate decreased in samples Nos. 3 and 4, ambiguous results were obtained in 50 cycle for qPCR. To ensure that there were no false positives, Nos. 3 and 4 were retested using ddPCR. The results showed that these samples were positive in ddPCR.

**Table 1:** Detection results for clinical samples

Groups		Quantitative real-time PCR			Droplet digital PCR	
		Positive	Negative	Suspicious	Positive	Negative
Single samples		40	10	0	40	10
Mixed samples	NO. 1	8	0	0	8	0
	NO. 2	8	0	0	8	0
	NO. 3	7	0	1	8	0
	NO. 4	6	0	2	8	0
Total		69	10	3	72	10
Positive rate%		84.1%			87.8%	

## Discussion

BoHV-1 is an economically significant viral infectious agent that is transmitted through semen via natural insemination and AI in cattle and buffalo. Therefore, routine monitoring of semen has been established worldwide. Transmission risk can be reduced by screening contaminated semen and discarding positive batches [21, 22]. Various test methods have been developed for the detection of BoHV-1 in bovine semen. Viral isolation is considered the gold standard, but this method requires cells to be cultured for a long time and does not suitable for low virus load, particularly in semen [23, 24]. Molecular diagnostic methods have been developed for rapid and sensitive detection of BoHV-1 in clinical samples. Reddy et al. used real-time PCR study for detection of BoHV-1 to study the shedding of BoHV-1 in extended bovine semen of cattle and buffaloes [25]. qPCR has been prescribed by OIE for detection of IBR-seropositive bulls for international trade. Although qPCR has played an important role in the diagnosis of BoHV-1, the detection methods would provide better diagnostic resources [26].

ddPCR technology is an improved PCR, this method based on limiting dilutions and Poisson statistics. Indeed, ddPCR has emerged as a high sensitive and precise method for detecting nucleic acid quantification in samples. Cray et al. optimized the multiplexed ddPCR method to quantify the ratio of chromosomes in frozen bovine sexed semen [27]. Because it is more sensitive than qPCR, it can detect target DNA at low concentrations. Therefore, this method has been used to diagnose variety of diseases and is especially useful for low viral loads, allowing more timely treatment and prevention of viral infections [28]. In addition, the ddPCR assay had significantly fewer false negatives and positives than qPCR in samples with low viral load. De-Falco et al. used ddPCR to detect BPV (bovine papillomavirus) DNA in blood samples for cows, and the positivity rate was significantly higher than that of qPCR [29]. de Oliveira et al. reported that ddPCR was able to detect low copy numbers of OvHV-2 in sheep and cattle [30]. de Oliveira et al. developed a novel RT-ddPCR method for the detection of FMDV RNA, and it was used to test 60 bovine samples from animals with suspected vesicular diseases that were previously tested by RT-qPCR [31].

During the development of the ddPCR method, we chose the gB gene to design the primers and probe. The gB gene is highly conserved among members of the *Herpesviridae* family and is frequently used as the target gene to detect BoHV-1 [32, 33]. The primers and probe used for ddPCR were the same as those used for qPCR, and we also developed a qPCR assay to validate the design. The results showed that the standard curves for BoHV-1 qPCR could be constructed, indicating that the primers and probe could be used for ddPCR. To optimize the annealing temperatures of the primers and probe for ddPCR, a range of annealing temperatures (from 56 to 62 °C) were compared. Based on the results, 57.2 °C was chosen for the ddPCR platform.

In this paper, we constructed a ddPCR assay, which was able to detect semen samples containing low concentrations of BoHV-1. We used serial 10-fold dilutions of plasmid DNA to evaluate the sensitivity and repeatability. The results indicated that ddPCR was more accurate than qPCR at lower concentrations. The ddPCR assay showed a detection limit of 4.45 copies per reaction in plasmid samples with good repeatability, whereas qPCR could detect only 4.45 × 10^2^ copies/μL. It was observed that the target DNA could not be detected at lower copy numbers by qPCR. In ddPCR technology, the reaction mixture can be divided into tens of thousands of water-in-oil droplets, and then, the reactions occur individually in these droplets [34]. Therefore, the viscosity of the aqueous phase interferes with droplet formation at higher DNA concentrations. This can lead to invalid results because there are too few negative droplets and low fluorescence amplitudes [35]. Therefore, we chose concentrations less than 4.45 × 10^4^ copies/μL to perform ddPCR for sensitivity and repeatability.

To evaluate the specificity of the BoHV-1 ddPCR assay, we chose vertically transmitted cattle pathogens, such as BTV, BVDV, *Brucella* and *M. bovis*. The method exhibited high specificity and no cross-reactivity signals with other above mentioned samples.

Subsequently, the technique was used for the detection of BoHV-1 in semen samples to evaluate the practicability of ddPCR and qPCR. The results demonstrated that ddPCR was more accurate than qPCR. In addition, we found that when the semen samples were independently tested, BoHV-1 DNA could be detected by both assays. However, the samples were mixed, leading to questionable qPCR results and positive ddPCR results. Usually, in large-scale detection of pathogens, the samples are mixed, which might decrease the concentration of the pathogen. On the other hand, the nucleic acid concentrations can be influenced by the DNA extraction method [36, 37]. Therefore, ddPCR is better suited for virus detection in mixed samples.

## Conclusion

We showed that the established ddPCR displayed good accuracy, repeatability and specificity. It could be used for accurate detection of BoHV-1 in semen samples. Therefore, it has the potential to compensate for the poor detection results obtained by qPCR. It can be used as an efficient detection method for BoHV-1 and for preventing disease transmission.

## Supplementary Information

**Additional file 1: Table S1.** Information on the primers and TaqMan probes. **Table S2.** Grouping of bovine semen samples.

**Additional file 2. **ddPCR of data.

**Additional file 3. **qPCR of data.

## Data Availability

The data supporting our findings of this article are included within the manuscript.

## Abbreviations

IBR: Infectious bovine rhinotracheitis
BoHV-1: Bovine alphaherpesvirus 1
AI: Artificial insemination
OIE: The World Health Organization for Animals
BTV: Bluetongue virus
BVDV: Bovine viral diarrhea virus
PCR: Polymerase chain reaction
qPCR: Quantitative real-time PCR
ddPCR: Droplet digital PCR
DNA: Deoxyribonucleic acid
RNA: Ribonucleic acid
BPV: Bovine papillomavirus

## References

[1] Hostnik P, Černe D, Mrkun J, Starič J, Toplak I (2021). Review of infections with bovine herpesvirus 1 in Slovenia. Front Vet Sci.

[2] De Brun L, Leites M, Furtado A, Campos F, Roehe P, Puentes R (2021). Field evaluation of commercial vaccines against infectious bovine rhinotracheitis (Ibr) virus using different immunization protocols. Vaccines (Basel).

[3] Petrini S, Iscaro C, Righi C (2019). Antibody responses to bovine alphaherpesvirus 1 (BoHV-1) in passively immunized calves. Viruses.

[4] Queiroz-Castro VLD, da Costa EP, Alves SVP, Machado-Neves M, Guimarães JD, Gomes LL, Domingos SV, Ribeiro CG, Caldas RT, Silva A (2019). Bovine herpesvirus 1 can cross the intact zona pellucida of bovine oocytes after artificial infection. PLoS One.

[5] Knopf L, Schwermer H, Stärk KD (2007). A stochastic simulation model to determine the sample size of repeated national surveys to document freedom from bovine herpesvirus 1 (BoHV-1) infection. BMC Vet Res.

[6] Mars MH, de Jong MC, van Maanen C, Hage JJ, van Oirschot JT (2000). Airborne transmission of bovine herpesvirus 1 infections in calves under field conditions. Vet Microbiol.

[7] Jolly WT (2020). National official assurance systems for international trade in animals and animal products, with reference to the standards of the world organisation for animal health. Rev Sci Tech.

[8] Holden SA, Butler ST (2018). Review: applications and benefits of sexed semen in dairy and beef herds. Animal.

[9] World organization for animal health (office international des Epizooties: OIE) (2021). Manual of Diagnostic Tests and Vaccines for Terrestrial Animals 2021.

[10] Da Silva N, Zardoya R, Santurde G (1995). Rapid and sensitive detection of the bovine viral diarrhea virus genome in semen. J Virol Methods.

[11] Revell SG, Chasey D, Drew TW, Edwards S (1988). Some observations on the semen of bulls persistently infected with bovine virus diarrhoea virus. Vet Rec.

[12] Klein D (2002). Quantification using real-time PCR technology: applications and limitations. Trends Mol Med.

[13] Taylor SC, Laperriere G, Germain H (2017). Droplet digital PCR versus qPCR for gene expression analysis with low abundant targets: from variable nonsense to publication quality data. Sci Rep.

[14] Rocha MA, Barbosa EF, Guimarães SE, Dias Neto E, Gouveia AM (1998). A high sensitivity-nested PCR assay for BHV-1 detection in semen of naturally infected bulls. Vet Microbiol.

[15] Oma VS, Klem T, Tråvén M, Alenius S, Gjerset B, Myrmel M, Stokstad M (2018). Temporary carriage of bovine coronavirus and bovine respiratory syncytial virus by fomites and human nasal mucosa after exposure to infected calves. BMC Vet Res.

[16] Park C, Lee J, Hassan ZU, Ku KB, Kim SJ, Kim HG, Park EC, Park GS, Park D, Baek SH, Park D, Lee J, Jeon S, Kim S, Lee CS, Yoo HM, Kim S (2021). Comparison of digital PCR and quantitative PCR with various SARS-CoV-2 primer-probe sets. J Microbiol Biotechnol.

[17] Chen B, Jiang Y, Cao X, Liu C, Zhang N, Shi D (2021). Droplet digital PCR as an emerging tool in detecting pathogens nucleic acids in infectious diseases. Clin Chim Acta.

[18] Kojabad AA, Farzanehpour M, Galeh HEG, Dorostkar R, Jafarpour A, Bolandian M, Nodooshan MM (2021). Droplet digital PCR of viral DNA/RNA, current progress, challenges, and future perspectives. J Med Virol.

[19] Verhaegen B, De Reu K, De Zutter L, Verstraete K, Heyndrickx M, Van Coillie E (2016). Comparison of droplet digital PCR and qPCR for the quantification of Shiga toxin-producing Escherichia coli in bovine feces. Toxins (Basel).

[20] Xie Z, Luo S, Xie L, Liu J, Pang Y, Deng X, Xie Z, Fan Q, Khan MI (2014). Simultaneous typing of nine avian respiratory pathogens using a novel GeXP analyzer-based multiplex PCR assay. J Virol Methods.

[21] Philpott M (1993). The dangers of disease transmission by artificial insemination and embryo transfer. Br Vet J.

[22] Rana SK, Kota SN, Samayam PN, Rajan S, Srinivasan VA (2011). Use of real-time polymerase chain reaction to detect bovine herpesvirus 1 in frozen cattle and buffalo semen in India. Vet Ital.

[23] Mahajan V, Banga HS, Deka D, Filia G, Gupta A (2013). Comparison of diagnostic tests for diagnosis of infectious bovine rhinotracheitis in natural cases of bovine abortion. J Comp Pathol.

[24] El-Mohamady RS, Behour TS, Rawash ZM (2020). Concurrent detection of bovine viral diarrhoea virus and bovine herpesvirus-1 in bulls' semen and their effect on semen quality. Int J Vet Sci Med.

[25] Reddy RVC, Putla B, Sarangi LN, Rana SK, Surendra KSNL, Ponnanna NM, Sharma GK (2020). Shedding of bovine alphaherpesvirus-1 in bovine extended frozen semen in Indian semen stations: a longitudinal analysis. Theriogenology.

[26] Sarangi LN, Naveena T, Rana SK, Surendra KSNL, Reddy RVC, Bajibabu P, Ponnanna NM, Sharma GK, Srinivasan VA (2018). Evaluation of a specialized filter-paper matrix for transportation of extended bovine semen to screen for bovine herpesvirus-1 by real-time PCR. J Virol Methods.

[27] Cray N, Wagner M, Hauer J, Roti Roti E (2020). Technical note: droplet digital PCR precisely and accurately quantifies sex skew in bovine semen. J Dairy Sci.

[28] Ren M, Lin H, Chen S, Yang M, An W, Wang Y, Xue C, Sun Y, Yan Y, Hu J (2018). Detection of pseudorabies virus by duplex droplet digital PCR assay. J Vet Diagn Investig.

[29] De Falco F, Corrado F, Cutarelli A, Leonardi L, Roperto S (2021). Digital droplet PCR for the detection and quantification of circulating bovine Deltapapillomavirus. Transbound Emerg Dis.

[30] de Oliveira TFP, Laguardia-Nascimento M, Xavier FG, Pinto CD, Ferreira LR, de Souza IDC, Hammerschmitt ME, Bianchi RM, Wronski JG, Etges RN, Rigon GM, Camargos MF, Júnior AVR, Fonseca Junior AA (2019). Quantification of ovine herpesvirus 2 by digital PCR in an outbreak of malignant catarrhal fever. Arch Virol.

[31] de Oliveira TFP, Fonseca AA, Camargos MF, Laguardia-Nascimento M, de Oliveira AM, Cottorello ACP, Goes-Neto A, Barbosa-Stancioli EF (2018). Development of a droplet digital RT-PCR for the quantification of foot-and-mouth virus RNA. J Virol Methods.

[32] Widen F, Goltz M, Wittenbrink N, Ehlers B, Banks M, Belak S (2001). Identification and sequence analysis of the glycoprotein B gene of porcine cytomegalovirus. Virus Genes.

[33] Chen R, Chen Q, Wu X, Che Y, Wang C, Wang L, Yan S, Zhou L (2020). Development of a TaqMan based real-time fluorescent quantitative PCR assay for detection of porcine cytomegalovirus in semen. Biomed Res Int.

[34] Zhang Y, Zhang Z, Wang Z, Wang Z, Wang C, Feng C, Yuan W, Lin X, Wu S (2019). Development of a droplet digital PCR assay for sensitive detection of porcine circovirus 3. Mol Cell Probes.

[35] Pinheiro LB, Coleman VA, Hindson CM, Herrmann J, Hindson BJ, Bhat S, Emslie KR (2012). Evaluation of a droplet digital polymerase chain reaction format for DNA copy number quantification. Anal Chem.

[36] Long S, Berkemeier B (2021). Development of a reverse transcription droplet digital PCR (RT-ddPCR) assay for sensitive detection of simian immunodeficiency virus (SIV). Virol J.

[37] Silva EC, Pelinca MA, Acosta AC, Silva DM, Gomes Filho MA, Guerra MM (2014). Comparative study of DNA extraction methodologies from goat sperm and its effects on polymerase chain reaction analysis. Genet Mol Res.

