# Supplementary material for: Development of a droplet digital PCR assay to detect bovine alphaherpesvirus 1 in bovine semen
Source: BMC Vet Res. 2022 Apr 2;18:125. doi: 10.1186/s12917-022-03235-2 (PMC8976375; doi:10.1186/s12917-022-03235-2)
Supplement: Supplementary file 1 — Additional file 1: Table S1. Information on the primers and TaqMan probes. Table S2. Grouping of bovine semen samples. [file 12917_2022_3235_MOESM1_ESM.doc]

Table S1. Information on the primers and TaqMan probes.

| Primers/Probe | Sequence（5’ to 3’） | Product  sizes (bp) |
| --- | --- | --- |
| BoHV-1-F  BoHV-1-R  BoHV-1-P | ACCTTTGTGAGCGACAGCC  TTGTAGCGCTCGCGGTAGAC  FAM-TCGCGTTGCAGAATGTGCCGCTGA-BHQ1 | 110 |

Table S2. Grouping of bovine semen samples.

| Groups | NO. 1 | NO. 2 | NO. 3 | NO. 4 |
| --- | --- | --- | --- | --- |
| Positive/Negative | 7:1 | 5:3 | 3:5 | 1:7 |
